# The evaluation of anaemia in an older primary care population: retrospective population-based study

**DOI:** 10.3399/bjgpopen17X101157

**Published:** 2017-10-04

**Authors:** David McCartney, Brian Shine, Deborah Hay, Daniel S Lasserson

**Affiliations:** 1 NIHR In Practice Fellow, Nuffield Department of Primary Care Health Sciences, Radcliffe Observatory Quarter, Oxford, UK; 2 Consultant in Chemical Pathology, Department of Clinical Biochemistry, Oxford University Hospitals Foundation Trust, Oxford, UK; 3 Consultant Haematologist, Department of Haematology, Oxford University Hospitals Foundation Trust, Oxford, UK; 4 Professor of Ambulatory Care, Institute of Applied Health Research, College of Medical and Dental Sciences, University of Birmingham, Birmingham, UK

**Keywords:** primary health care, general practice, anemia, geriatrics, diagnosis

## Abstract

**Background:**

Anaemia is common in older people and the identification of potentially reversible haematinic deficiencies relies on appropriate investigation, often undertaken in primary care.

**Aim:**

To determine the laboratory prevalence of anaemia, the types of anaemia observed, and the biochemical and haematological investigations undertaken to characterise any associated haematinic abnormality in older primary care patients.

**Design & setting:**

A retrospective primary care based study of patients aged >65 years undergoing a full blood count in Oxfordshire, UK between 1 January 2012 and 31 December 2013.

**Method:**

Consecutive patients aged >65 years with a full blood count were identified retrospectively from a laboratory database. Patient demographics, number of blood tests and additional laboratory investigations requested were recorded. World Health Organisation (WHO) criteria were used to define anaemia.

**Results:**

In total 151 473 full blood counts from 53 890 participants were included: 29.6% of patients were anaemic. The majority had a normocytic anaemia (82.4%) and 46.0% of participants with anaemia had no additional investigations performed. The mean haemoglobin was lower in the anaemic group that underwent further investigation than those who did not (Hb 10.68 g/dl versus 11.24 g/dl, *P*<0.05): 33.2 % of patients with a microcytic anaemia (mean cell volume <80) did not have any markers of iron status measured.

**Conclusion:**

A large proportion of older adults in primary care with a recent blood test are anaemic, the majority with a normocytic anaemia, with evidence of inadequate investigation. Those with lower haemoglobin are more likely to be further investigated. Further work is needed to understand the approach to anaemia in older adults in primary care.

## How this fits in

Anaemia is thought to be common in older adults with previous estimates suggesting prevalence of around 10%. It is increasingly recognised that anaemia is associated with a wide range of adverse outcomes including increased rates of hospitalisation, mortality, and cognitive decline. This analysis of a large dataset demonstrated a large proportion of people were anaemic and many did not undergo further blood tests to investigate possible causes of anaemia or associated haematinic deficiencies. These results suggest that more patients with anaemia should be tested further to determine possible causes and to guide management.

## Introduction

Anaemia is a common clinical problem in older populations.^1^ It is well recognised that haemoglobin decreases with advancing age^2^ and is often observed in the context of multimorbidity. Most recent estimates of anaemia in older populations have suggested an overall prevalence of 10%^3^ although estimates are necessarily dependent on definitions used and populations studied. The WHO categorise men with haemoglobin <13.0 g/dL and women with haemoglobin <12.0 g/dL as anaemic,^4^ although there are no age-specific reference values. These cut-offs are typically used to highlight abnormal laboratory results in a clinical setting. In older populations where levels below these thresholds are commonly found it is unclear whether these thresholds are appropriate or whether age-adjusted thresholds would be preferable.^3,5,6^

Evidence demonstrates independent associations of anaemia with a wide range of adverse outcomes including increased mortality, hospitalisation,^7^ and poorer quality of life.^8^ However, it is unclear to what extent correcting anaemia in older patients reverses the observed adverse associations and improves outcomes. There is mixed evidence from randomised controlled trials^9–13^ and a recent systematic review showed that in adults there was no improvement in mortality with oral or intravenous iron supplementation (although there was no subgroup analysis by age).^14^

The identification and further characterisation of types of anaemia often relies on investigations undertaken by GPs in primary care. Further characterisation is considered necessary to identify potentially treatable and reversible haematinic deficiencies such as B12, folate, or iron deficiency. There is little consensus as to the level of anaemia that requires further evaluation in an older population^15^ and little guidance on sequence of investigation. Furthermore, characterisation of haematinic abnormalities and deficiencies can be challenging in older adults with results difficult to interpret in the context of inflammation^16^ and potentially diagnostic abnormalities in mean cell volume (MCV) and mean cell haemoglobin (MCH) not always observed in older adults due to associated chronic disease.^17^

There are few studies examining the behaviour of GPs in the further investigation of abnormal haematological results. Two previous observational studies have reported suboptimal endoscopic investigation of iron deficiency anaemia by GPs^18,19^ but there has been little assessment of pragmatic repeat testing and haematinic evaluation in undifferentiated anaemia. The objective of this study was to determine the laboratory prevalence of anaemia in older primary care patients, and to describe the diagnostic investigations undertaken by GPs in a large older adult community cohort in order to better understand current practice and identify areas for further research and improvement.

## Method

All full blood count requests from general practice between 1 January 2012 and 31 December 2013 (24-month interval period) for adults aged >65 years at the time of the request were identified from the Oxford University Hospitals NHS Trust (OUH) laboratory database. Linked biochemistry results for first-line investigations also requested from general practice were extracted: these included ferritin, transferrin saturation, serum transferrin, iron, serum B12, serum folate, C-reactive protein, and creatinine (standardised to isotope dilution mass spectrometry methods) and estimated glomerular filtration rate (eGFR). If additional biochemical investigations were not requested at the same time point as the full blood count on which anaemia was identified, results from additional biochemical investigations were extracted if requested at any timepoint within the 24-month study period. If these were requested on more than one occasion the earliest request was used in the analysis. Demographic data (date of birth and sex) were included. All requests from outside general practices were excluded from this analysis; for example, community hospitals. During the study period the OUH laboratory were using the Sysmex XE analyser.

The study was conducted as a service evaluation using de-identified patient data and the OUH Research and Development Department confirmed that ethical approval was not required.

## Statistical analysis

The WHO definition of anaemia was used to estimate prevalence.^4^ Normocytic anaemia was defined as a mean cell volume (MCV) between 80 and 100 fL with a microcytic anaemia being <80 fL and a macrocytic anaemia being >100 fL. A cut off of 30 μg/L defined low serum ferritin. The main outcome measures were the proportion of individuals with haemoglobin below the reference definition at any time point over a 2-year period defined by each age group (65–74, 75–84, 85–94, and ≥95 years) and proportion of patients in which further biochemical investigations were undertaken focused on determining an underlying cause. The distributions of haemoglobin among different age groups were determined. All analyses were undertaken using IBM SPSS Statistics (version 22).

## Results

**Figure 1.**
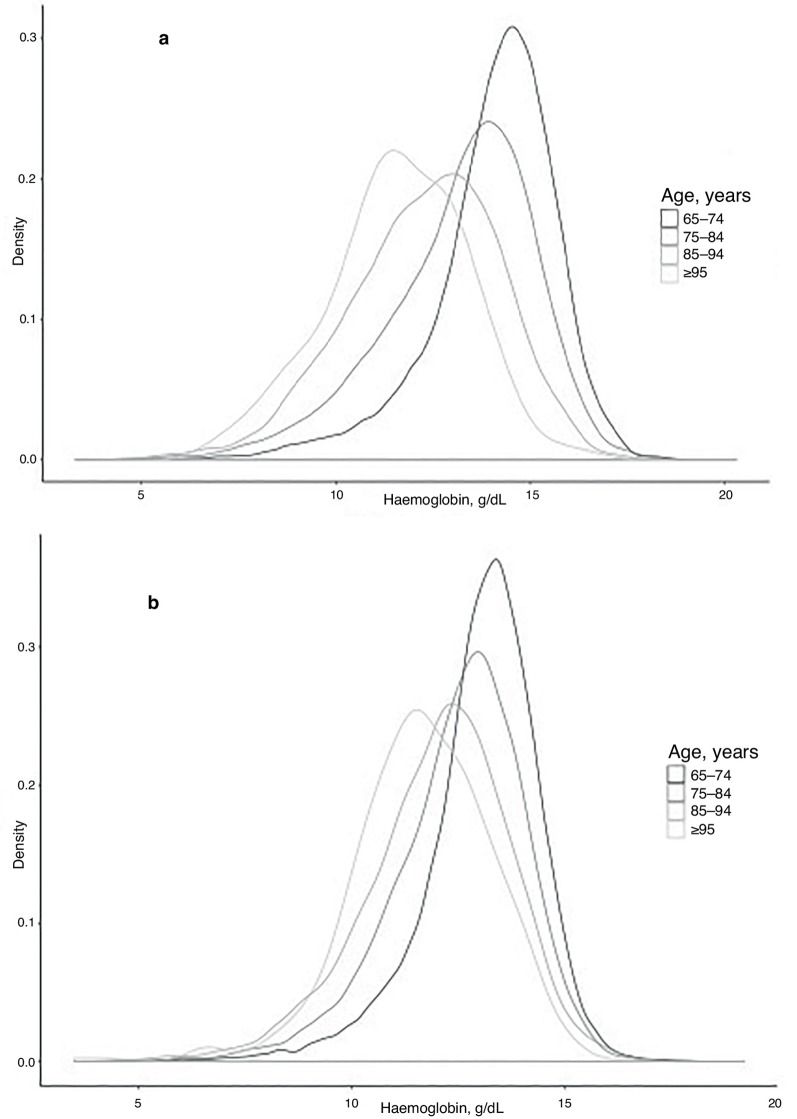
Kernel density plots to demonstrate the distribution of Hb, and changes with age decile in men (**a**) and women (**b**).

In total, 151 473 full blood counts were analysed from 53 890 older adults (44.7% male) aged ≥65 years over a 24-month period. This represents 47.8% of the estimated population aged >65 years in Oxfordshire CCG (2015 estimates). Table 1 shows the number of full blood counts per patient. There were 44.0% of patients who had a single full blood count during the 24-month period, while 23.5% of patients had two full blood counts. The maximum number of full blood counts over this period was 53. The overall mean haemoglobin of those aged >65 years was 13.02 g/dl (standard deviation 1.79). There was a significant difference between the mean haemoglobin (Hb) for men and women aged >65 years (13.59g/dl versus 12.63 g/dl, *P*<0.001). There was a significant progressive decline in mean Hb with increasing age decile in both men and women (Table 2), with distributions shown in Figure 1.

**Table 1.** Number of full blood counts

Full blood count, *n*	Patients, *n*	Patients, %
1	23 700	44.0
2	12 641	23.5
3	7062	13.1
4	3943	7.3
≥5	6544	12.1

**Table 2.** Mean haemoglobin (Hb) in men and women.

	Men			Women		
Age, years	Patients, *n*	Mean Hb	Standard deviation	Patients, *n*	Mean Hb	Standard deviation
65–74	11 944	14.07	1.65	13 204	13.05	1.38
75–84	8742	13.18	1.95	10 459	12.50	1.57
85–94	3203	12.26	1.98	5650	12.00	1.72
≥95	185	11.56	1.79	503	11.67	1.64

**Figure 2.**
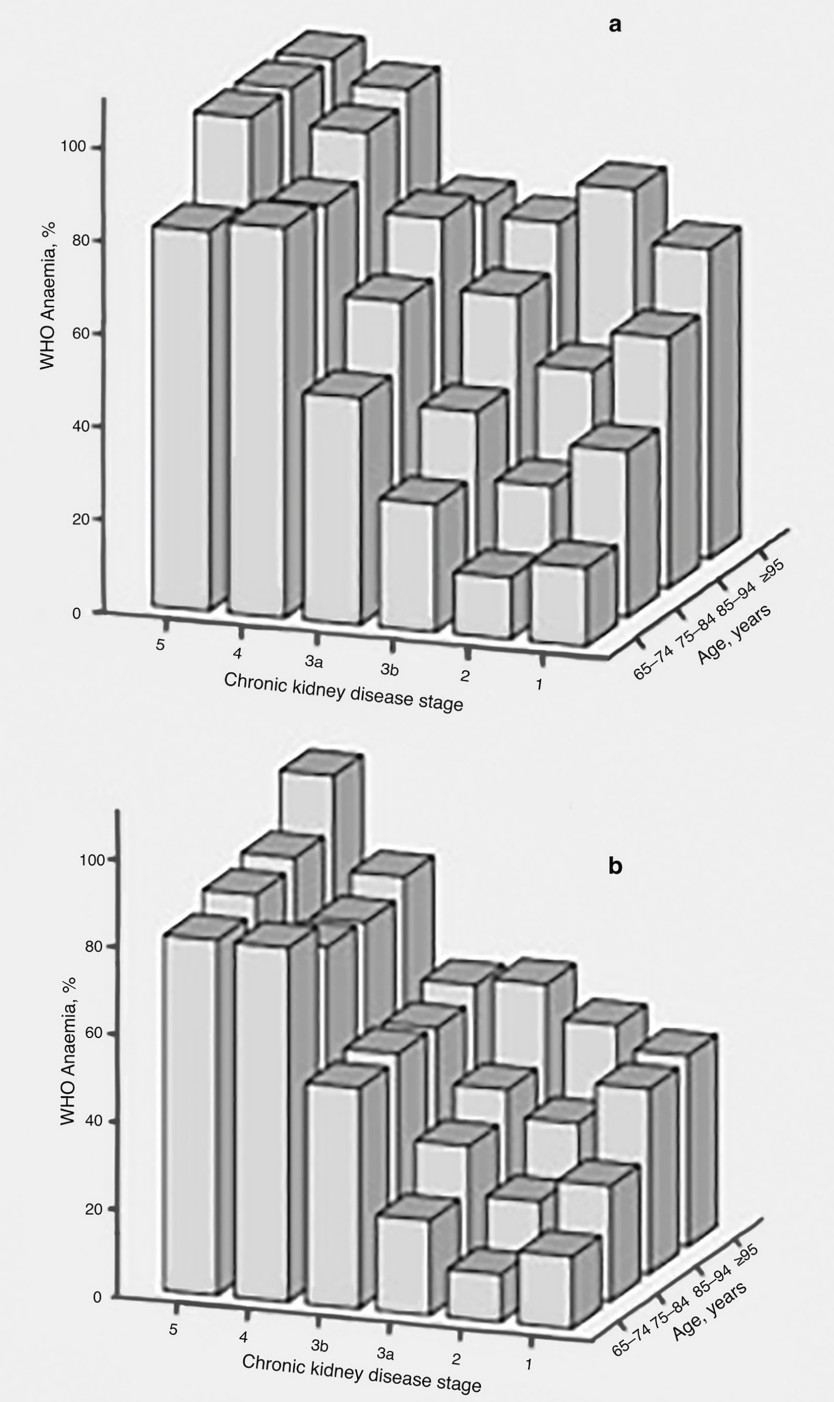
Distribution of anaemia according to age and CKD status in men (**a**) and women (**b**).

Overall, 15 950 (29.6%) of patients were anaemic according the WHO criteria (32.0% of men and 27.7% of women). The proportion with anaemia increased with age and with worsening chronic kidney disease (CKD) stage for both men and women as shown in Figure 2, in particular demonstrating an increase in anaemia from stage 3a to 3b CKD. The majority of the 15 950 anaemic patients had a normocytic anaemia (82.4%). Patients with a low MCV represented only 7.3% of this cohort. The remaining 10.3% had a macrocytic anaemia.

In total, 29.1% of patients who were anaemic according to WHO criteria (4648 individuals) did not have a repeat full blood count over a 24-month period. Within the 24-month interval sampled, 55.4% of patients (8850 individuals) had at least one additional test to evaluate for potential underlying haematinic deficiencies (this includes serum B12, serum folate, red cell folate, iron, ferritin, serum transferrin, and transferrin saturation). The proportion of individuals who had at least one additional test was similar in both men and women. The mean Hb was lower in the anaemic group where at least one further test had been done than in the anaemic group who did not have any further investigation. This was the case for both men (Hb 10.96 g/dl versus 11.56 g/dl, *P*<0.001) and women (Hb 10.45 g/dl versus 10.91 g/dl, *P*<0.001)

Overall, 7503 patients (47.0%) with anaemia according to WHO criteria had a serum ferritin measured. This proportion was higher in those with an MCV <80 fl (66.8% with a serum ferritin value) and lower in those with a normal MCV (45.4%)

Of those that did have a ferritin measured, 2288 (30.5%) had a low serum ferritin (<30 μg/l). For those in whom the ferritin was >30 μg/l, only 1983 (38.0%) had a simultaneous measurement of C-reactive protein (CRP) to understand if this normal value represented iron sufficiency or was raised due to an acute or chronic inflammatory response.^20^ Where ferritin was raised and CRP was also measured this was raised above 10 in 835 cases (42.1%) and above 30 in 331 cases (17.1%). The mean CRP was significantly higher in the anaemic population than in the non-anaemic population (27.93 versus 9.63, *P*<0.001). There was no significant difference in mean ferritin between the anaemic and non-anaemic populations, even when those with coexisting inflammation suggested by a CRP value >10 were excluded (132.69 versus 135.78 *P* = 0.38). The majority of patients (6551) who underwent sampling of serum ferritin also had a serum transferrin saturation measured. Of these, 3887 individuals (59.3%) had a low transferrin saturation (<20%).

## Discussion

### Summary

Anaemia is prevalent among an older population with a recent blood test. Around one in three patients in this cohort are identified as anaemic by WHO criteria. These data suggest that there may be potential underinvestigation in the over 65s. Just under one-third of patients identified as anaemic did not have a repeat full blood count over a 2-year period. Furthermore, a significant number of patients with anaemia did not have any test of haematinic deficiency to identify any potentially treatable causes: less than half of those with an identified anaemia had a serum ferritin measured.

Those that undergo further testing have an average haemoglobin lower than those that do not. These findings suggest that those with what may be considered a mild anaemia are those that are underinvestigated.

There are several possible reasons that this may be the case. First, it may be that a mild anaemia is being considered insignificant in the over 65s and that further investigation is not necessary. Indeed, there has been much debate about whether the WHO thresholds are appropriate for an older population, in light of the fact that they were developed in the 1960s based on standard deviations of a population mean derived from a population aged <65 years. Previous studies have proposed both higher and lower alternative thresholds.^21^ Most recent linked data from the Health Survey for England demonstrated significantly reduced survival in men with haemoglobin below WHO thresholds, supporting their continued use.^22^

Other possible explanations for lack of haematinic testing include an empirical approach to treatment with the avoidance of further investigation, a strategy which may be appropriate depending on clinical circumstances or avoidance of investigation because of expected difficulties in interpretation of results due to associated disease. Haematinic tests (in particular those directed at identifying iron deficiency anaemia) have been shown to have a low sensitivity and specificity in older people.^23^

## Strengths and limitations

The main strengths of this study are the large general older population and inclusion of consecutive samples over a 2-year period, thus allowing a detailed and robust assessment of the parameters of interest. Over 50 000 patients aged >65 years were included in the analysis and as far as the authors are aware this is the largest community cohort to date evaluating anaemia prevalence and investigations in a primary care population selected for testing.

There are some limitations within this work. Data extracted from a general laboratory database includes those that have had blood tests for many reasons. This is likely to bias this study towards those with chronic disease. The estimates of prevalence will overestimate true prevalence in an older adult population. The definition of anaemia may also have overestimated prevalence: anaemia was defined on the basis of a single abnormal haemoglobin result; a more conservative approach would require a confirmatory sample. The rationale was that a large number of individuals without any repeat full blood count (whether confirmatory or not) would have been excluded from further analysis. In the assessment of subsequent investigation and follow-up, a 24-month window period was used. Some individuals may have been investigated outside this window period (for example, if blood tests were taken before or after date cut offs) and therefore this work may overestimate those not undergoing further haematinic testing. The possibility that some practices may have used point-of-care testing is noted, and results of repeat samples may therefore not be available in laboratory data. However, additional testing for the causes of anaemia currently require the use of the central laboratory as there are no routinely available point-of-care tests for haematinics.

## Comparison with existing literature

A previous systematic review looked at the prevalence of anaemia in the population aged >60 years^1^ concluding that prevalence estimates vary widely (2.9–61% in men and 3.3–41% in women), depending on definitions used and populations studied. The use of traditional markers of anaemia such as MCV to categorise anaemia in older patients has previously been questioned.^20^ This study's data show that the majority of patients have a normocytic anaemia and using MCV alone is therefore likely to miss many cases of iron deficiency anaemia and therefore a full assessment of iron status is essential.

The literature also acknowledges that, due to the common overlap of iron deficiency and inflammation in older patients, the sensitivity of other markers used to identify iron deficiency anaemia such as ferritin and transferrin saturation may be reduced.^24^ These findings demonstrate that a significant proportion of participants with anaemia and a normal ferritin have evidence of co-existing inflammation (suggested by a raised CRP), potentially reducing the diagnostic value of this marker.

Rimon *et al* ^23^ recommend the use of the transferrin receptor-ferritin index as a diagnostic marker of iron deficiency anaemia. Their work demonstrated that this index has a better sensitivity for iron deficiency than ferritin alone, although in older adults a low ferritin in the presence of a raised CRP has high predictive value for anaemia,^25^ suggesting that a low ferritin is a useful rule in test but a normal ferritin should not be used to exclude an iron deficiency anaemia particularly if there is evidence of any degree of inflammation.

Previous studies have examined the extent to which iron deficiency anaemia identified by GPs prompts gastrointestinal investigations with endoscopy.^18,26^ These studies both demonstrated low rates of investigation (43% and 31%), although no studies that we are aware of have reported haematinic testing and laboratory evaluation following an abnormal haemoglobin result. The current data did not allow assessment of subsequent hospital and secondary care investigation but the findings are consistent with previous research showing many individuals with anaemia are not fully evaluated.

## Implications for research and practice

This study demonstrates the potential to underdiagnose and reliably exclude iron deficiency in an older population which is associated with an inadequate assessment of haematinics. This is of importance, given the associations between iron deficiency anaemia and gastrointestinal pathology: in one study over half of older patients with an identified iron deficiency anaemia have a gastrointestinal tract abnormality.^27^

The future use of novel biomarkers may aid more accurate diagnosis iron deficiency. Further work is needed to establish the sensitivity of other markers of iron deficiency in older adults. Serum hepcidin has shown promise in distinguishing different types of anaemia in certain populations^28,29^ and also in predicting response to iron supplementation, while serum transferrin receptor assay has been shown to have a good sensitivity in older adults.^23^

Future research should be directed at improving the diagnostic pathway for anaemia in an older adult population. This includes understanding why some individuals remain uninvestigated, and evaluating the use of novel biomarkers. Furthermore, greater understanding about potential reversibility, impact of treatment on quality of life, morbidity and other outcomes measures are essential. Given the prevalence of anaemia and the increasing age of our population, widely-adopted mechanisms for improved management of anaemia in people aged >65 years are essential.

**Table 3:** Percentage of patients investigated.

	*N*	TSat, %	Ferritin, %	Paired C-reactive protein, %	B12, %	Folate, %
Microcytic	1167	59.0	66.8	20.7	51.6	47.0
Normocytic	13 236	39.6	45.4	16.5	43.3	38.6
Macrocytic	1542	40.2	46.4	18.5	51.6	46.4
